# Supplementary material for: Gene Flow and Diversification in Himalopsyche martynovi Species Complex (Trichoptera: Rhyacophilidae) in the Hengduan Mountains
Source: Biology (Basel). 2021 Aug 23;10(8):816. doi: 10.3390/biology10080816 (PMC8389565; doi:10.3390/biology10080816)
Supplement: Supplementary file 1 [file biology-10-00816-s001.zip › File S1.pdf]

Table S1. List of all the samples.

Sample ID	SRA Accession	Lab Code	Species	Identifier	Sex	Life Stage	Collectors	Collection Date	Country	Lat	Lon	Elev	Institution Storing	Reference
**4**	SRR15101121	I19058_4	*H.viteceki*	Anna E. Hjalmarsson	M	Adult	Chen, Hjalmarsson, Li	30.7.2013	China	28.2934	99.153	3306	SGN Frankfurt	This study
**5**	SRR15101120	I19059_5	*H.viteceki*	Anna E. Hjalmarsson	M	Adult	Chen, Hjalmarsson, Li	30.7.2013	China	28.2934	99.153	3306	SGN Frankfurt	This study
**14**	SRR15101118	I19067_14	*H. martynovi sensu stricto*	Anna E. Hjalmarsson	M	Adult	Saldaitis	06.10.2010	China	29.8667	102.3	2100	SGN Frankfurt	This study
**12**	SRR15101117	I19065_12	*H. martynovi sensu stricto*	Hans Malicky	M	Adult	Saldaitis	05.7.2010	China	32.9167	103.4	3500	SGN Frankfurt	This study
**16**	SRR15101116	I19068_16	*H. martynovi sensu stricto*	Hans Malicky	M	Adult	Floriani & Saldaitis	23.8.2014	China	31.483210	102.493400	3400	SGN Frankfurt	This study
**11**	SRR15101115	I19064_11	*H. cf. martynovi*	Hans Malicky	M	Adult	Floriani	08.10.2011	China	30.0667	101.417	3611	SGN Frankfurt	This study
**13**	SRR15101114	I19066_13	*H. martynovi sensu stricto*	Anna E. Hjalmarsson	M	Adult	Floriani & Saldaitis	27.8.2014	China	33.175770	104.321200	2900	SGN Frankfurt	This study
\		SPHIM424-17	*H.martynovi*	Hans Malicky	M	Adult	Floriani	08.10.2011	China	30.0667	101.417	3611	Malicky, Lunz	Hjalmarsson et al. 2018
\		SPHIM425-17	*H.martynovi*	Hans Malicky	M	Adult	Saldaitis	05.7.2010	China	32.9167	103.4	3500	Malicky, Lunz	Hjalmarsson et al. 2018
\		SPHIM427-17	*H.martynovi*	Anna E. Hjalmarsson	M	Adult	Saldaitis	06.10.2010	China	29.8667	102.3	2100	Malicky, Lunz	Hjalmarsson et al. 2018
\		SPHIM428-17	*H.martynovi*	Anna E. Hjalmarsson	M	Adult	Saldaitis	14.7.2009	China	30.0667	101.433	3500	Malicky, Lunz	Hjalmarsson et al. 2018
\		SPHIM429-17	*H.martynovi*	Hans Malicky	M	Adult	Floriani & Saldaitis	22.8.2014	China	30.4667	101.633	3500	Malicky, Lunz	Hjalmarsson et al. 2018
\		SPHIM430-17	*H.martynovi*	Hans Malicky	M	Adult	Floriani & Saldaitis	23.07.2011	China	33.1333	103.717	3000	Malicky, Lunz	Hjalmarsson et al. 2018
**7**	SRR15101113	I19061_7	*H.epikur*	Hans Malicky	M	Adult	Chen, Hjalmarsson, Li	07.8.2013	China	28.5389	99.8174	3609	SGN Frankfurt	This study
**6**	SRR15101112	I19060_6	*H.epikur*	Anna E. Hjalmarsson	M	Adult	Chen, Hjalmarsson, Li	07.8.2013	China	28.5389	99.8174	3609	SGN Frankfurt	This study
**8**	SRR15101111	I19062_8	*H.epikur*	Anna E. Hjalmarsson		Larva	Tachamo Shah, Shah, Jaehnig	10.2011	China	27.632556	99.368167	2500	SGN Frankfurt	This study
\		SPHIM405-17	*H.epikur*	Hans Malicky	M	Adult	Chen, Hjalmarsson, Li	07.8.2013	China	28.5389	99.8174	3609	SGN Frankfurt	Hjalmarsson et al. 2018
\		HspXC0104FAH0697_677	*H.epikur*	Anna E. Hjalmarsson	F	Adult	Chen, Hjalmarsson, Li	09.8.2013	China	29.116	100.033	4154	SGN Frankfurt	
\		SPHIM415-17	*H.epikur*	Anna E. Hjalmarsson	M	Adult	Chen, Hjalmarsson, Li	09.8.2013	China	28.4095	99.7764	3040	SGN Frankfurt	Hjalmarsson et al. 2018
\		SPHIM439-17	*H.epikur*	Hans Malicky	M	Adult	Saldaitis	10.4.2004	China	28.5333	99.8167	3500	Malicky, Lunz	Hjalmarsson et al. 2018
\		LZ78_F	*H.epikur*	Anna E. Hjalmarsson	F	Adult	Floriani & Saldaitis	19.6.2015	China	29.2833	100.083	4050	SGN Frankfurt	
**3**	SRR15101119	I19057_3	*H.immodesta*	Anna E. Hjalmarsson	M	Adult	Chen, Hjalmarsson, Li	23.7.2013	China	26.0218	99.888	2728	SGN Frankfurt	This study

*SGN Frankfurt: Senckenberg Research Institute and Natural History Museum

Table S2. Information of the 1–Kite transcriptomes for probe design.

**Genus**	**Species**	
Platycentropus	*radiatus*	INSbttTARAAPEI-9
Cheumatopsyche	*sp.*	INSbttTCRAAPEI-92
Chimarra	*sp.*	INSbttTDRAAPEI-118
Leptocerus	*americanus*	INSbttTERAAPEI-126
Nectopsyche	*albida*	INSbttTFRAAPEI-171
Psychomyia	*flavida*	INSbttTHRAAPEI-15
Ptilostomis	*sp.*	INSbttTHRAAPEI-17
Glyphotaelius	*pellucidus*	INSbusTBDRAAPEI-17
Phryganea	*grandis*	INShauTBBRAAPEI-22
Hydroptilidae	*sp.*	INSnfrTBJRAAPEI-8
Rhyacophila	*fasciata*	INSjdsTBSRAAPEI-9
Apatania	*incerta*	INShkeTAARAAPEI-94
Lepidostoma	*togatum*	INShkeTACRAAPEI-8
Agapetus	*hessi*	INShkeTADRAAPEI-9
Diplectrona	*sp.*	INShkeTBWRAAPEI-17
Bombyx	*mori*	Proteom

Table S3. Best model selected by jModelTest2 based on the concatenated sequence.

	Model	f(a)	f(c)	f(g)	f(t)	kappa	titv	Ra	Rb	Rc	Rd	Re	Rf	pInv	gamma
AIC	GTR+I+G	0.31	0.18	0.19	0.32	0.00	0.00	0.902	2.665	1.261	0.806	3.465	1.000	0.86	0.90
BIC	GTR+I+G	0.31	0.18	0.19	0.32	0.00	0.00	0.902	2.665	1.261	0.806	3.465	1.000	0.86	0.90
AICc	GTR+I+G	0.31	0.18	0.19	0.32	0.00	0.00	0.902	2.665	1.261	0.806	3.465	1.000	0.86	0.90
DT	GTR+I+G	0.31	0.18	0.19	0.32	0.00	0.00	0.902	2.665	1.261	0.806	3.465	1.000	0.86	0.90

Table S4. Gene flow frequency classified by species.

	Direction of gene flow	Frequency
1	*H. viteceki* / *H. viteceki* <–> *H.* cf. *martynovi*	1
2	*H. martynovi* s.s. / *H. martynovi* s.s. <–> *H.* cf. *martynovi*	11
3	*H. martynovi* s.s. –> *H.* cf. *martynovi*	2
4	*H. epikur* / *H. epikur* <–> *H. martynovi* s.s.	10
5	none	1731

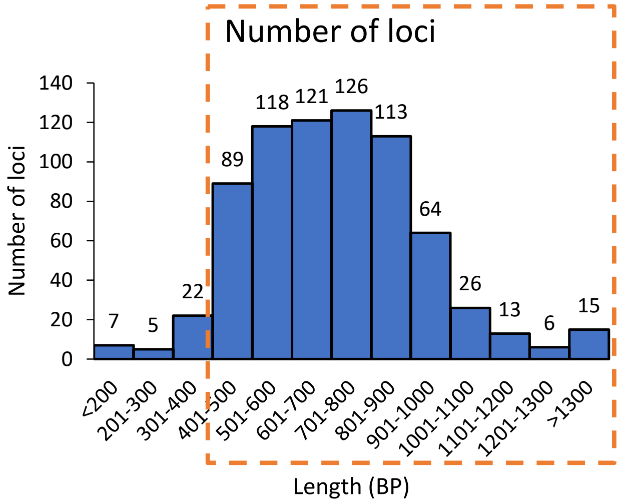

Figure S1. Length distribution of anchored hybrid enrichment loci after removing gaps and removing loci cover clusters less than 75% individuals. The mean sequence length was 716 bp and the standard deviation was 237 bp. The dashed orange box shows the loci with a length higher than 400 bp which were used for further analyses, in total 691 loci, 509113 bp overall, of which 65.8% were identical, GC content was 37.6%, gap (including ambiguous) percentage was 4%. The number above the bar shows the total number of loci for each length range.
